# Supplementary material for: Neural circuits for long-term water-reward memory processing in thirsty Drosophila
Source: Nat Commun. 2017 May 15;8:15230. doi: 10.1038/ncomms15230 (PMC5440665; doi:10.1038/ncomms15230)
Supplement: Supplementary Information — Supplementary Figures and Supplementary Note 1 [file ncomms15230-s1.pdf]

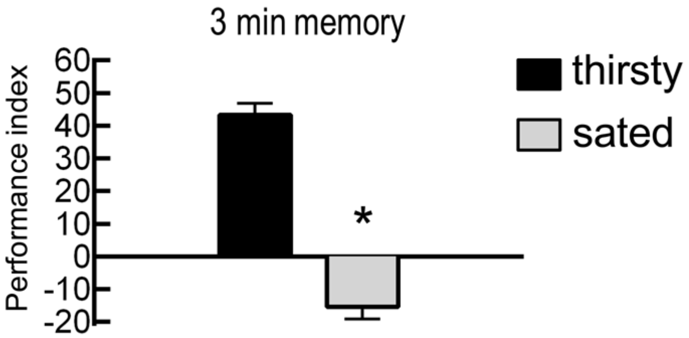

Supplementary Figure 1. Flies form water-reward memory only in the thirsty state

Thirsty but not sated wild-type flies form robust 3 min memory. For the thirsty group, the flies were water-deprived for 16 h before conditioning. For the sated group, the 16-h water-deprived flies were allowed to drink water for 30 min before conditioning. Each value represents mean ± SEM (N = 8 for each bar). **P* < 0.05; *t*-test.

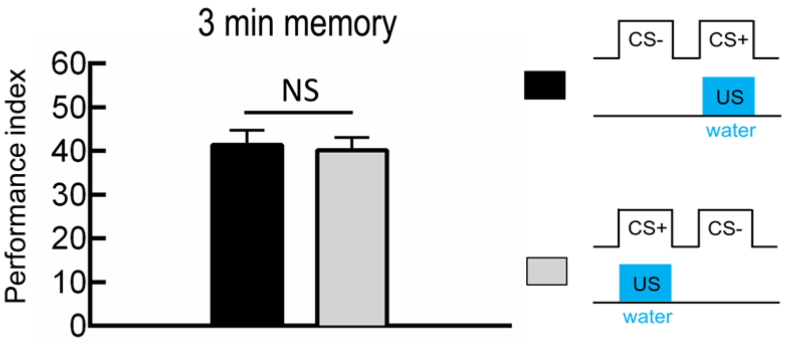

Supplementary Figure 2. Different training procedures produce similar memory performances

Water paired with the second odor presentation during training (black bar; CS-/CS+) produced similar 3 min memory performance compared to that in the group in which water was paired with the first odor during training (gray bar: CS+/CS-). Each value represents mean ± SEM (N = 8 for each bar). NS, not significant ($P > 0.05$); *t*-test.

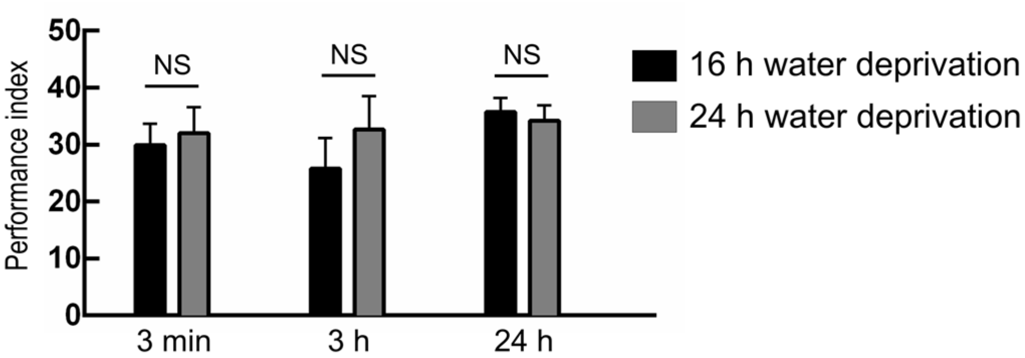

Supplementary Figure 3. Water deprivation for 16 h and 24 h before training produces similar memory performances

Both 16- and 24-h water-deprivation states before conditioning produced similar learning, STM, and LTM performances in wild-type flies. Each value represents mean ± SEM (N = 8, 8, 6, 6, 8, 8 from left to right bars). NS, not significant ($P > 0.05$); *t*-test.

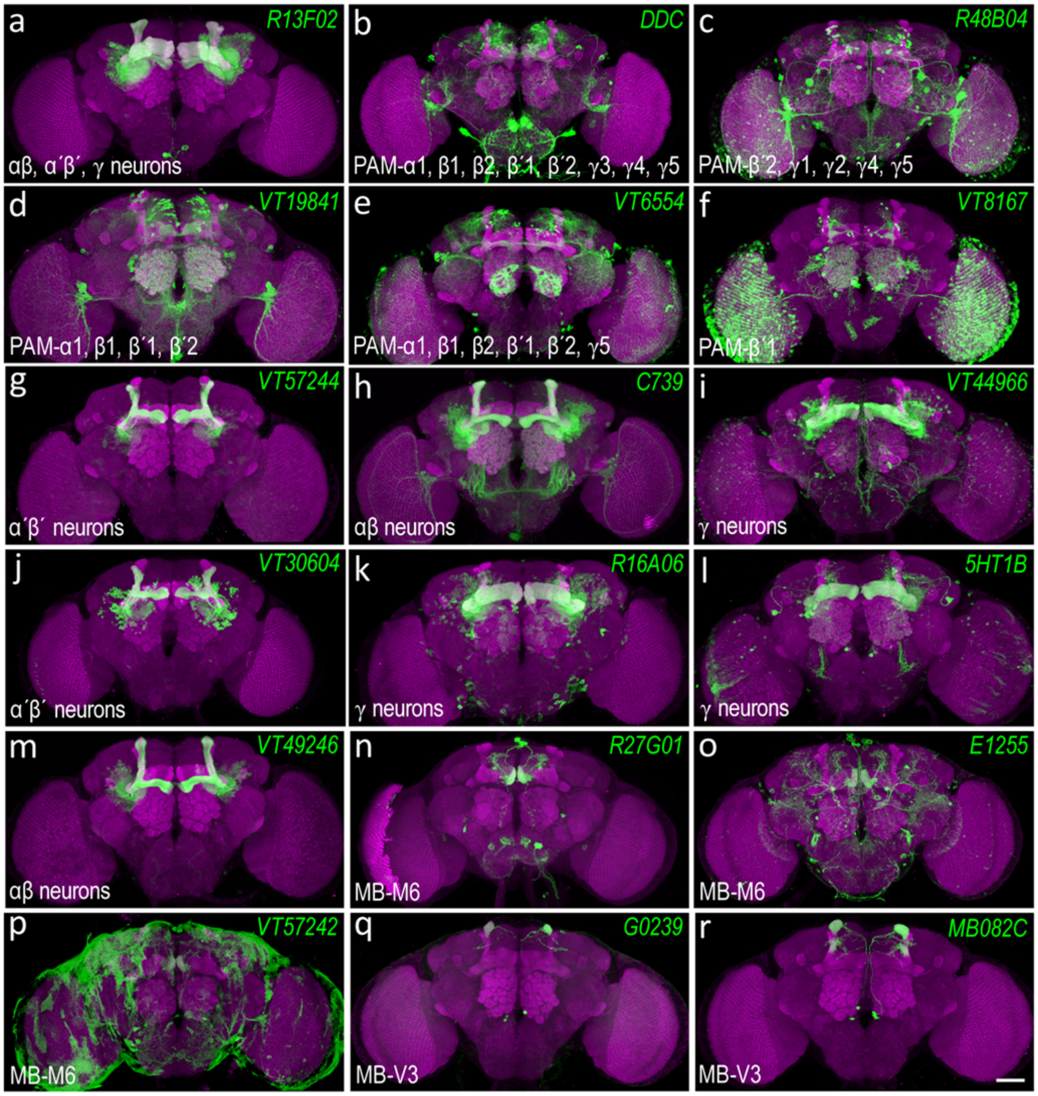

Supplementary Figure 4. GFP expression patterns driven by GAL4 lines used in this study

(**a**) All MB neurons under *R13F02-GAL4* expression. (**b–f**) PAM neurons under *DDC-GAL4* (**b**), *R48B04-GAL4* (**c**), *VT19841-GAL4* (**d**), *VT6554-GAL4* (**e**), and *VT8167-GAL4* (**f**) expression. (**g**) MB α′β′ neurons under VT*57244-GAL4* expression. (**h**) MB αβ neurons under *C739-GAL4* expression. (**i**) MBs γ neurons under *VT44966-GAL4* expression. (**j**) MB α′β′ neurons under *VT30604-GAL4* expression. (**k,l**) MBs γ neurons under *R16A06-GAL4* (**k**) and *5HT1B-GAL4* (**l**) expression. (**m**) MB αβ neurons under *VT49246-GAL4* expression. (**n–p**) MB-M6 neurons under *R27G01-GAL4* (**n**), *E1255-GAL4* (**o**), and *VT57242-GAL4* (**p**) expression. (**q,r**) MB-V3 neurons under *G0239-GAL4* (**q**) and *MB082C-GAL4* (**r**) expression. Brain neuropils were counterstained with anti-DLG immunostaining (magenta). Scale bar represents

50 µm. Genotypes: (1) *+/UAS-mCD8::GFP; R13F02-GAL4/UAS-mCD8::GFP*, (2) *DDC-GAL4/+; +/UAS-mCD8::GFP; +/UAS-mCD8::GFP*, (3) *+/UAS-mCD8::GFP; R48B04-GAL4/UAS-mCD8::GFP*, (4) *+/UAS-mCD8::GFP; VT19841-GAL4/UAS-mCD8::GFP*, (5) *+/UAS-mCD8::GFP; VT6554-GAL4/UAS-mCD8::GFP*, (6) *+/UAS-mCD8::GFP; VT8167-GAL4/UAS-mCD8::GFP*, (7) *+/UAS-mCD8::GFP; VT57244-GAL4/UAS-mCD8::GFP*, (8) *C739-GAL4/UAS-mCD8::GFP; +/UAS-mCD8::GFP*, (9) *+/UAS-mCD8::GFP; VT44966-GAL4/UAS-mCD8::GFP*, (10) *+/UAS-mCD8::GFP; VT30604-GAL4/UAS-mCD8::GFP*, (11) *+/UAS-mCD8::GFP; R16A06-GAL4/UAS-mCD8::GFP*, (12) *5HT1B-GAL4/UAS-mCD8::GFP; +/UAS-mCD8::GFP*, (13) *+/UAS-mCD8::GFP; VT49246-GAL4/UAS-mCD8::GFP*, (14) *+/UAS-mCD8::GFP; R27G01-GAL4/UAS-mCD8::GFP*, (15) *E1255-GAL4/+; +/UAS-mCD8::GFP; +/UAS-mCD8::GFP*, (16) *+/UAS-mCD8::GFP; VT57242-GAL4/UAS-mCD8::GFP*, (17) *G0239-GAL4/UAS-mCD8::GFP; +/UAS-mCD8::GFP*, and (18) *R40B08-GAL4AD/UAS-mCD8::GFP; R23C06-GAL4BD/UAS-mCD8::GFP*.

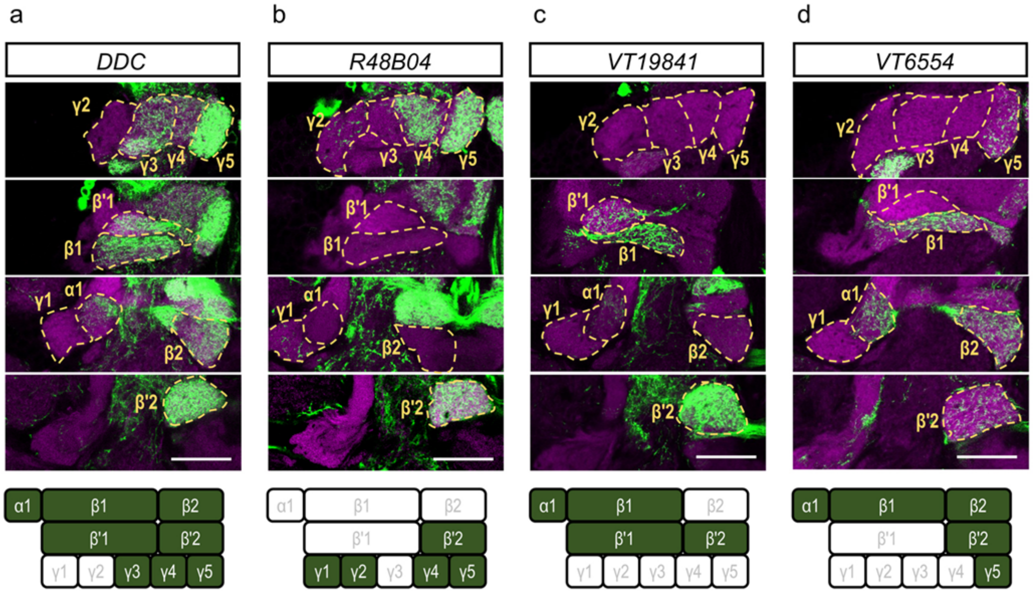

Supplementary Figure 5. The expression patterns of PAM-GAL4 lines used in this study

Horizontal MB lobes innervation of *DDC-GAL4* (**a**), *R48B04-GAL4* (**b**), *VT19841-GAL4* (**c**), and *VT6554-GAL4* (**d**) fly brains were revealed using a *UAS-mCD8::GFP;UAS-mCD8::GFP* reporter. The MB neuropil structures were labeled with DLG antibody (magenta). Scale bars represent 20 μm. Genotypes: (1) *DDC-GAL4/+; +/UAS-mCD8::GFP; +/UAS-mCD8::GFP/+*, (2) *+/UAS-mCD8::GFP; R48B04-GAL4/UAS-mCD8::GFP*, (3) *+/UAS-mCD8::GFP; VT19841-GAL4/UAS-mCD8::GFP*, (4) *+/UAS-mCD8::GFP; VT6554-GAL4/UAS-mCD8::GFP*.

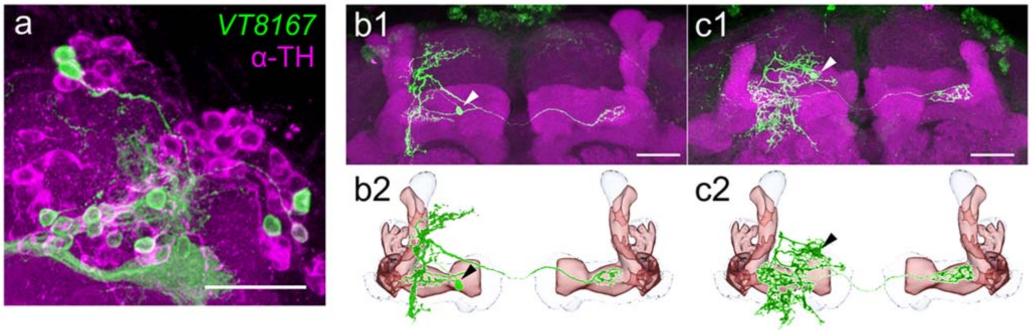

Supplementary Figure 6. Morphology and innervation patterns of individual dopaminergic PAM-β´1 neurons in relation to MB volume model

(**a**) The somas of PAM-β´1 are TH-immunopositive. The brain was labeled with anti-TH antibody (magenta). Scale bars represent 20 μm. (**b1**–**c2**) Gross morphology and innervation patterns of a single PAM-β´1 neuron in the context of an MB volume model. Individual image of a single PAM-β´1 neuron was derived from FLP-out labeling of *VT8167-GAL4*. Arrowheads indicate the somas of PAM-β´1. The MB neuropil structures were labeled with DLG antibody (magenta). Genotype: *hs-flp/+; +/+; VT8167-GAL4/UAS >rCD2,y+>mCD8::GFP*. Scale bars represent 20 μm.

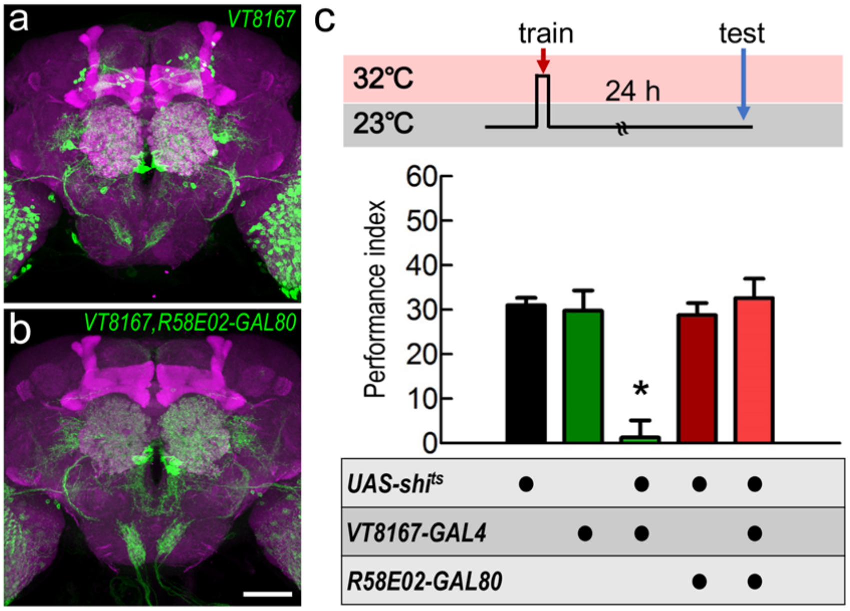

Supplementary Figure 7. Water-reward LTM requires reinforcing dopamine from PAM-β′1 neurons

(**a**) The expression pattern of *VT8167-GAL4*-driven GFP in PAM-β′1 neurons in the adult fly brain. (**b**) *VT8167-GAL4*-driven GFP expression in the dopaminergic PAM-β′1 neurons was removed using an overlapping *R58E02-GAL80* transgene. Scale bar, 50 μm. Genotypes: (1) *+/UAS-mCD8::GFP; VT8167-GAL4/+* and (2) *+/UAS-mCD8::GFP; VT8167-GAL4/R58E02-GAL80*. (**c**) The LTM defect in the *VT8167-GAL4 > UAS-shi*^{ts} flies were rescued by removing the expression in dopaminergic PAM-β′1 neurons using an overlapping *R58E02-GAL80* transgene. Each value represents mean ± SEM (N = 8 for each bar). **P* < 0.05; ANOVA followed by Tukey's test. Genotypes: (1) *+/UAS-shi*$^{ts(JFRC100)}$*; +/+*, (2) *+/+; VT8167-GAL4/+*, (3) *+/UAS-shi*$^{ts(JFRC100)}$*; VT8167-GAL4/+*, (4) *+/UAS-shi*$^{ts(JFRC100)}$*; R58E02-GAL80/+*, and (5) *+/UAS-shi*$^{ts(JFRC100)}$*; VT8167-GAL4/R58E02-GAL80*.

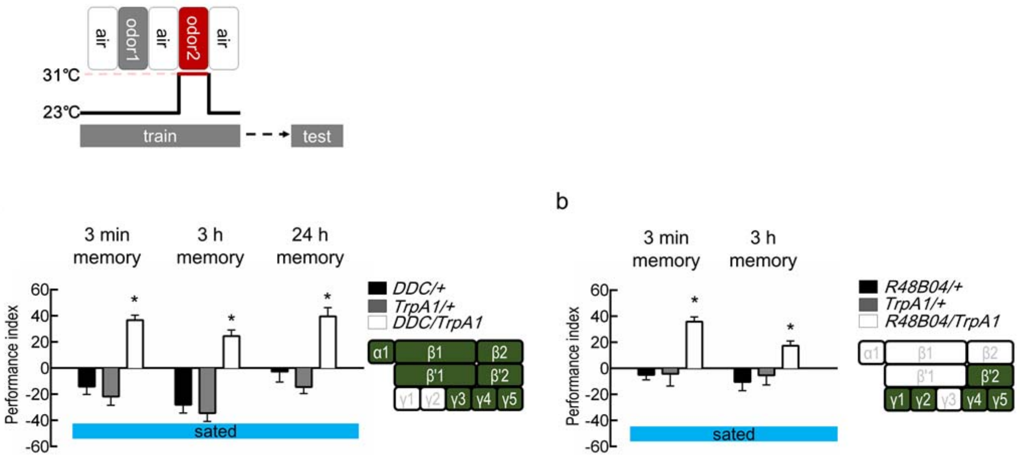

Supplementary Figure 8. Significant implanted memories are formed in water-sated *DDC-GAL4/UAS-TrpA1* and *R48B04-GAL4/UAS-TrpA1* flies

(**a**) Pairing the odor presentation with TrpA1-mediated activation of the *DDC* dopaminergic neurons during memory acquisition resulted in significant learning, STM, and LTM in water-sated flies. Genotypes: (1) *DDC-GAL4/+; +/+; +/+*, (2) *+/+; +/UAS-TrpA1*, and (3) *DDC-GAL4/+; +/+; +/UAS-TrpA1*. Each value represents mean ± SEM (N =12, 12, 12, 6, 6, 6, 8, 8, and 8 from left to right bars). **P* < 0.05; ANOVA followed by followed by Tukey's test. (**b**) Pairing the odor presentation with TrpA1-mediated activation of the *R48B04* dopaminergic neurons during memory acquisition resulted in significant learning and STM in water-sated flies. Genotypes: (1) *+/+; R48B04-GAL4/+*, (2) *+/+; +/UAS-TrpA1*, and (3) *+/+; R48B04-GAL4/UAS-TrpA1*. Each value represents mean ± SEM (N = 6, 6, 6, 8, 8, and 8 from left to right bars). **P* < 0.05; ANOVA followed by Tukey's test.

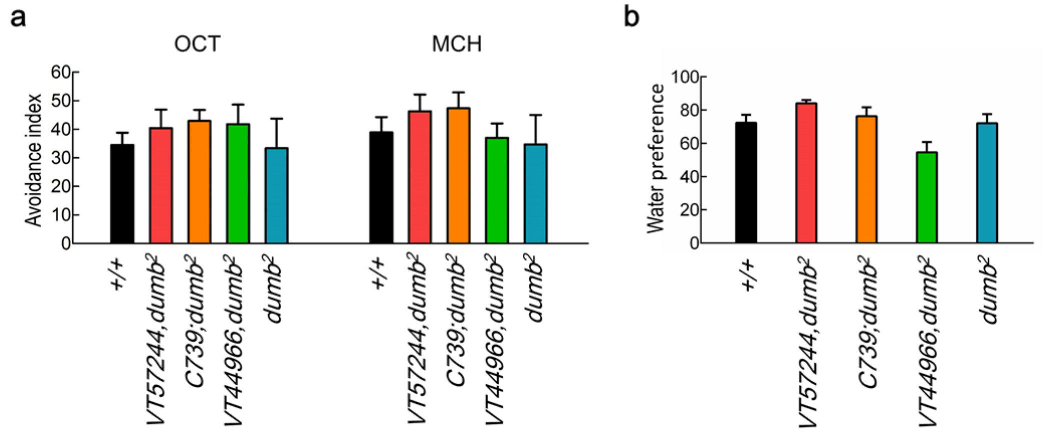

Supplementary Figure 9. Normal odor acuity and water preference in thirsty *dumb*^{2} flies

(**a**–**b**) Odor acuity and water preference controls for the DopR1 rescue experiment. All DopR1 mutant flies showed normal odor acuity to OCT and MCH and water preference compared with wild-type controls (+/+). Genotypes: (1) +/+*;* +/+, (2) +/+*; VT57244-GAL4,dumb*^{2}*/VT57244-GAL4,dumb*^{2}, (3) *C739-GAL4/C739-GAL4; dumb*^{2}*/dumb*^{2}, (4) +/+*; VT44966-GAL4,dumb*^{2}*/VT44966-GAL4,dumb*^{2}, (5) +/+*; dumb*^{2}*/dumb*^{2}. Each value represents mean ± SEM (N = 8, 8, 8, 8, 6, 8, 8, 10, 8, and 6 form left to right bars in (**a**), $P > 0.05$, ANOVA; N = 8, 8, 8, 7, and 8 form left to right bars in (**b**), $P > 0.05$, ANOVA).

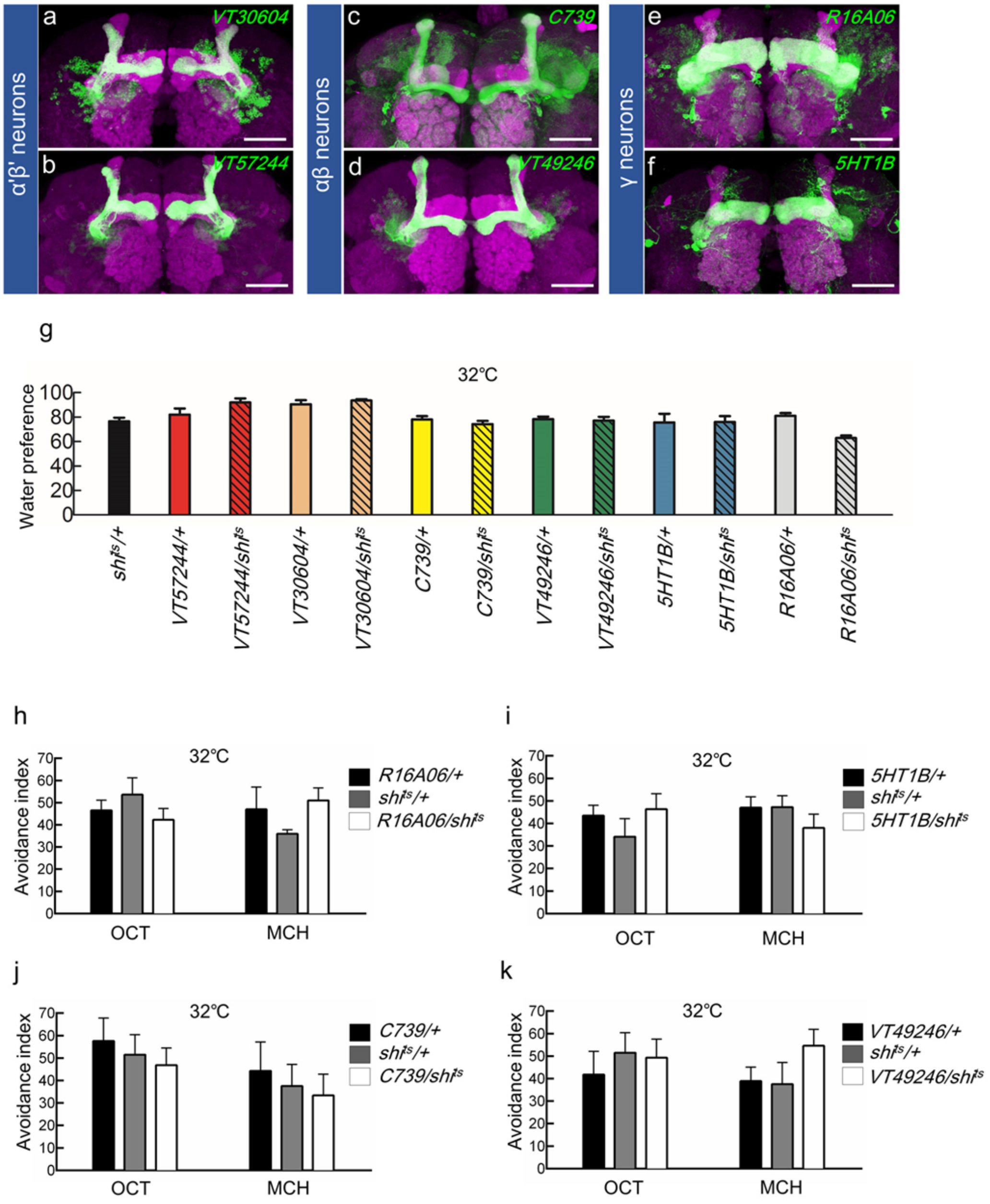

Supplementary Figure 10. Expression patterns and behavioral controls of MB-GAL4 lines used in this study

(**a**–**f**) GFP expression patterns driven by GAL4 lines used in this study. (**a**, **b**) MB α′β′ neurons under *VT30604-GAL4* (**a**) and *VT57244-GAL4* (**b**) expression. (**c**, **d**) MB αβ neurons under *C739-GAL4* (**c**) and *VT49246-GAL4* (**d**) expression. (**e**, **f**) MB γ neurons under *R16A06-GAL4* (**e**) and *5HT1B-GAL4* (**f**) expression. The MB neuropil structures were labeled with anti-DLG antibody (magenta). Scale bars represent 20 µm. (**g**) The *VT57244-GAL4* > *UAS-shi*^{ts}, *VT30604-GAL4* > *UAS-shi*^{ts}, *C739-GAL4* > *UAS-shi*^{ts}, *VT49246-GAL4* > *UAS-shi*^{ts}, *5HT1B-GAL4* > *UAS-shi*^{ts}, and *R16A06-*

GAL4 > *UAS-shi*^{ts} flies showed a normal water-preference response at 32°C restrictive temperature compared with their internal controls. Each value represents mean ± SEM (N = 30, 6, 6, 6, 6, 16, 16, 16, 16, 8, 8, 8, and 8 from left to right bars). $P > 0.05$; ANOVA. (**h**–**k**) The *R16A06-GAL4* > *UAS-shi*^{ts}, *5HT1B-GAL4* > *UAS-shi*^{ts}, *C739-GAL4* > *UAS-shi*^{ts}, and *VT49246-GAL4* > *UAS-shi*^{ts} flies showed normal odor acuity to OCT or MCH at 32°C restrictive temperature compared with their internal controls. Each value represents mean ± SEM (N = 8 for each bar in (**h**), $P > 0.05$, ANOVA; N = 8 for each bar in (**i**), $P > 0.05$, ANOVA; N = 10, 8, 8, 9, 8, and 8 from left to right bars in (**j**), $P > 0.05$; ANOVA; N = 10, 8, 8, 10, 8, and 8 from left to right bars in (**k**), $P > 0.05$, ANOVA).

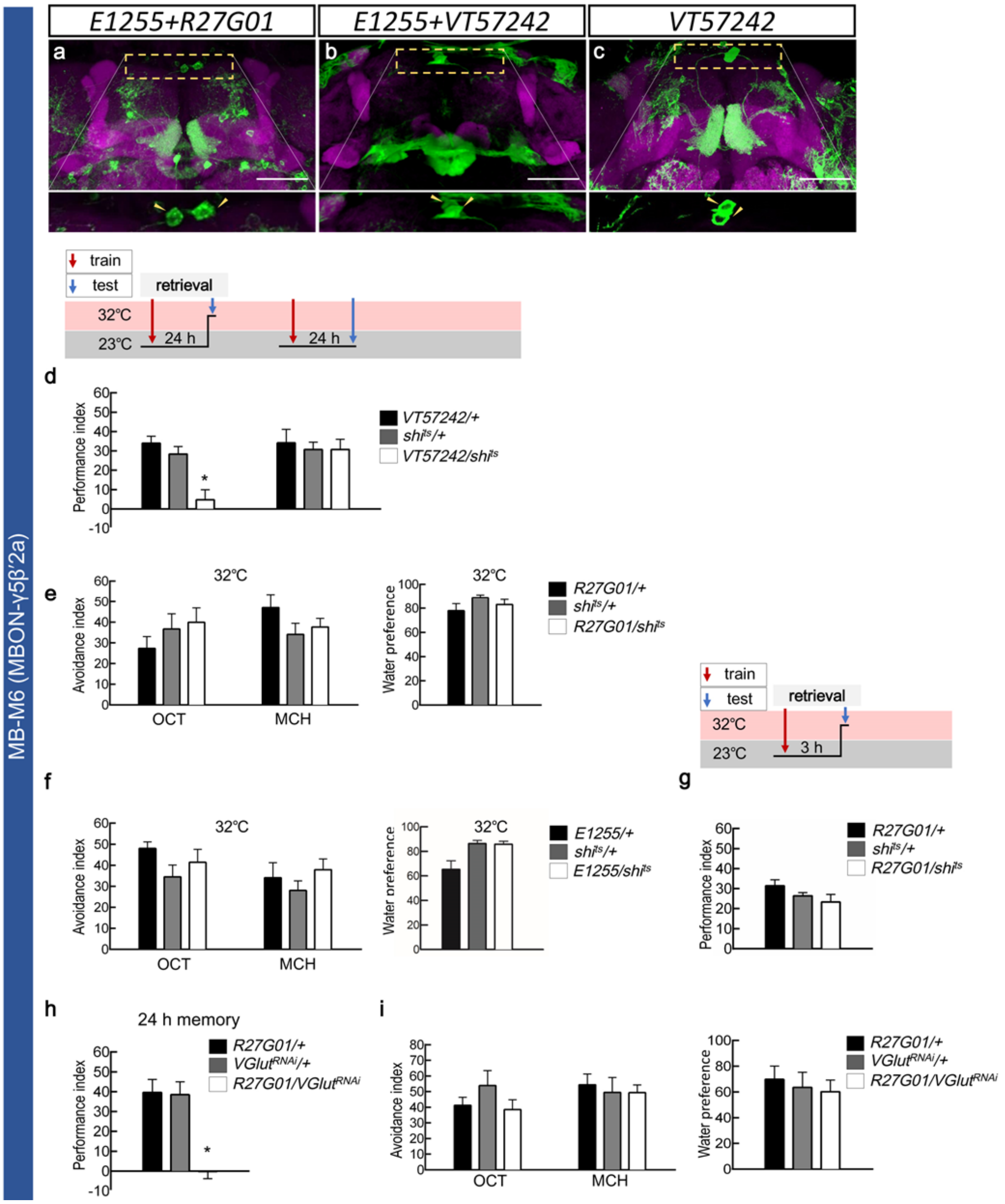

Supplementary Figure 11. Expression patterns and behavioral controls of MB-M6 GAL4 lines used in this study

(**a**) *E1255-GAL4* and *R27G01-GAL4* drive reporter gene expression in the same MB-M6 neurons. Arrowheads indicate the somas of MB-M6. The MB neuropil structures were labeled with anti-DLG antibody (magenta). Scale bars represent 20 μm. (**b**) *E1255-GAL4* and *VT57242-GAL4* drive reporter gene expression in the same MB-M6 neurons. The MB neuropil structures were labeled with anti-DLG antibody (magenta). Arrowheads indicate the somas of MB-M6. Scale bar represents 20 μm. (**c**) The expression pattern of *VT57242-GAL4*. The MB neuropil structures were labeled with

anti-DLG antibody (magenta). Arrowheads indicate somas. Scale bar represents 20 µm. (**d**) Blocking the output from MB-M6 during retrieval in flies carrying *VT57242-GAL4 > UAS-shi*^{ts} disrupted LTM. Each value represents mean ± SEM (N = 8 for each bar). *$P < 0.05$; ANOVA followed by Tukey's test. (**e**) Normal odor acuity to OCT or MCH and normal water preference at 32°C restrictive temperature in *R27G01-GAL4 > UAS-shi*^{ts} flies. Each value represents mean ± SEM (N = 8 for each bar in odor avoidance, $P > 0.05$, ANOVA; N = 8 for each bar in water preference assays, $P > 0.05$, ANOVA). (**f**) Normal odor acuity to OCT or MCH and normal water preference at 32°C restrictive temperature in *E1255-GAL4 > UAS-shi*^{ts} flies. Each value represents mean ± SEM (N = 7, 6, 8, 7, 8, and 7 from left to right bars in odor avoidance assay, $P > 0.05$, ANOVA; N = 8 for each bar in water preference assay, $P > 0.05$, ANOVA). (**g**) Blocking synaptic output from MB-M6 neurons during retrieval did not affect STM. Each value represents mean ± SEM (N = 10 for each bar). $P > 0.05$; ANOVA. (**h**) Constitutive silencing of *VGlut* in MB-M6 neurons disrupted LTM. Each value represents mean ± SEM (N = 8 for each bar). *$P < 0.05$; ANOVA followed by Tukey's test. (**i**) Genetic silencing of *VGlut* in MB-M6 neurons did not affect odor acuity to OCT or MCH, or water preference in thirsty flies. Each value represents mean ± SEM (N = 6 for each bar in odor avoidance, $P > 0.05$, ANOVA; N = 6 for each bar in water preference assays, $P > 0.05$, ANOVA).

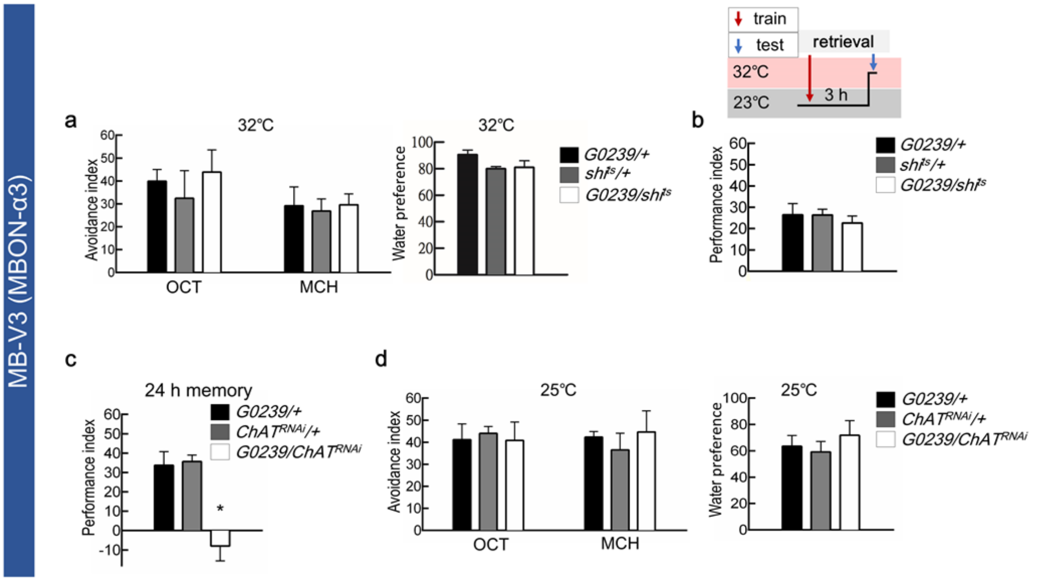

Supplementary Figure 12. Behavioral controls of MB-V3 GAL4 lines used in this study

(**a**) Normal odor acuity to OCT or MCH and normal water preference at 32°C restrictive temperature in *G0239-GAL4* > *UAS-shi*^{ts} flies. Each value represents mean ± SEM (N = 8, 7, 7, 6, 6, and 7 form left to right bars in odor avoidance assay, $P > 0.05$, ANOVA; N = 6 for each bar in water preference assay, $P > 0.05$, ANOVA). (**b**) Blocking synaptic output from MB-V3 neurons during retrieval did not affect STM. Each value represents mean ± SEM (N = 8 for each bar). $P > 0.05$; ANOVA. (**c**) Constitutive silencing of *ChAT* in MB-V3 neurons affected LTM. Each value represents mean ± SEM (N = 8 for each bar). *$P < 0.05$; ANOVA followed by Tukey's test. (**d**) Genetic silencing of *ChAT* in MB-V3 neurons did not affect odor acuity to OCT or MCH, or water preference. Each value represents mean ± SEM (N = 6 for each bar in odor avoidance, $P > 0.05$, ANOVA; N = 6 for each bar in water preference assays, $P > 0.05$, ANOVA).

Supplementary Note 1

Genotypes

Figure 1. Genotypes: (1) +/+, (2) *ppk28* mutant, (3) *P{EP}*^{cerG6085}, (4) *PBac{WH}teq*^{f01792}, (5) *rsh*^{1}, (6) *+/UAS-mCD8-GFP; R13F02-GAL4/UAS-mCD8::GFP*, (7) +/+; *R13F02-GAL4/+*, (8) *UAS-dCREB2-b/+; +/tub-GAL80*^{ts}, and (9) *UAS-dCREB2-b/+; R13F03-GAL4/tub-GAL80*^{ts}.

Figure 2. Genotypes: (1) *DDC-GAL4/+; +/+; +/+*, (2) +/+; *+/UAS-shi*^{ts}, (3) *DDC-GAL4/+; +/+; +/UAS-shi*^{ts}, (4) +/+*; R48B04-GAL4/+*, (5) +/+*; R48B04-GAL4/UAS-shi*^{ts}, (6) +/+; *VT19841-GAL4/+*, (7) +/+; *VT19841-GAL4/UAS-shi*^{ts}, (8) +/+*; VT6554-GAL4/+*, (9) +/+*; VT6554-GAL4/UAS-shi*^{ts}, (10) *+/UAS-mCD8::GFP; VT8167-GAL4/UAS-mCD8::GFP*, (11) +/+*; VT8167-GAL4/+*, and (12) +/+*; VT8167-GAL4/UAS-shi*^{ts}.

Figure 3. Genotypes: (1) +/+*; +/UAS-shi*^{ts}, (2) +/+; *VT8167-GAL4/+*, (3) +/+*; VT8167-GAL4/UAS-shi*^{ts}, (4) +/+*; VT6554-GAL4/+*, (5) +/+*; VT6554-GAL4/UAS-shi*^{ts}.

Figure 4. Genotypes: (1) *DDC-GAL4/+; +/+; +/+*, (2) +/+*; +/UAS-TrpA1*, (3) *DDC-GAL4/+; +/+; +/UAS-TrpA1*, (4) +/+*; R48B04-GAL4/+*, (5) +/+*; R48B04-GAL4/UAS-TrpA1*, (6) +/+*; VT8167-GAL4/+*, (7) +/+*; VT8167-GAL4/UAS-TrpA1*, (8) +/+*; +/+*, (9) +/+*; VT57244-GAL4,dumb*^{2}*/VT57244-GAL4,dumb*^{2}, (10) *C739-GAL4/C739-GAL4; dumb*^{2}*/dumb*^{2}, (11) +/+*; VT44966-GAL4,dumb*^{2}*/VT44966-GAL4,dumb*^{2}, (12) +/+*; dumb*^{2}*/dumb*^{2}.

Figure 5. Genotypes: (1) *DDC-GAL4/+; +/+; +/UAS-GCaMP6m* and (2) *+/+; VT8167-GAL4/UAS-GCaMP6m*.

Figure 6. Genotypes: (1) +/+*; VT30604-GAL4/*+, (2) +/+*; +/UAS-shi*^{ts}, (3) +/+*; VT30604-GAL4/UAS-shi*^{ts}, (4) +/+*; VT57244-GAL4/*+, (5) +/+*; VT57244-GAL4/UAS-shi*^{ts}, (6) +/+*; R16A06-GAL4/*+, (7) +/+*; R16A06-GAL4/UAS-shi*^{ts}, (8) *5HT1B-GAL4/+; +/+*, (9) *5HT1B-GAL4/+; +/UAS-shi*^{ts}, (10) +/+*; VT49246-GAL4/*+, (11) +/+*; VT49246-GAL4/UAS-shi*^{ts}, (12) *C739-GAL4/+; +/+*, and (13) *C739-GAL4/+; +/UAS-shi*^{ts}.

Figure 7. Genotypes: (1) *+/UAS-mCD8::GFP; R27G01-GAL4/UAS-mCD8::GFP*, (2) *E1255-GAL4/+; +/ UAS-mCD8::GFP; +/UAS-mCD8::GFP*, (3) +/+*; R27G01-GAL4/*+, (4) +/+*; +/UAS-shi*^{ts}, (5) +/+*; R27G01-GAL4/UAS-shi*^{ts}, (6) *E1255-GAL4/*+; +/+*;* +/+, (7) *E1255-GAL4/+; +/+; +/UAS-shi*^{ts}, (8) *+/UAS-VGlut*^{RNAi}*; +/tub-GAL80*^{ts}, (9) *+/UAS-VGlut*^{RNAi}*; R27G01-GAL4/tub-GAL80*^{ts}, (10) *G0239-GAL4/UAS-mCD8::GFP; +/UAS-mCD8::GFP*, (11) *R40B08-GAL4AD/UAS-mCD8::GFP; R23C06-GAL4BD/UAS-mCD8::GFP*, (12) *G0239-GAL4/+; +/+*, (13) *G0239-GAL4/+; +/UAS-shi*^{ts}, (14) *R40B08-GAL4AD /+; R23C06-GAL4BD/*+, (15) *R40B08-GAL4AD/+; R23C06-GAL4BD/UAS-shi*^{ts}, (16) *G0239-GAL4/+; +/tub-GAL80*^{ts}, (17) +/+*; +/UAS-ChAT*^{RNAi}, and (18) *G0239-GAL4/+; tub-GAL80*^{ts}*/UAS-ChAT*^{RNAi}.
